# Transcranial alternating current stimulation at 10 Hz modulates response bias in the Somatic Signal Detection Task

**DOI:** 10.1016/j.ijpsycho.2018.12.001

**Published:** 2019-01

**Authors:** Matt Craddock, Ekaterini Klepousniotou, Wael El-Deredy, Ellen Poliakoff, Donna Lloyd

**Affiliations:** a School of Psychology, University of Lincoln, LN6 7TS, UK; b School of Psychology, University of Leeds, Leeds LS2 9JT, UK; c Centro de Investigación y Desarrollo en Ingeniería en Salud, Universidad de Valparaiso, Chile; d Division of Neuroscience and Experimental Psychology, University of Manchester, M13 9PL, UK

**Keywords:** Somatosensation, Alpha oscillations, Transcranial alternating current stimulation, Signal detection theory

## Abstract

Ongoing, pre-stimulus oscillatory activity in the 8–13 Hz alpha range has been shown to correlate with both true and false reports of peri-threshold somatosensory stimuli. However, to directly test the role of such oscillatory activity in behaviour, it is necessary to manipulate it. Transcranial alternating current stimulation (tACS) offers a method of directly manipulating oscillatory brain activity using a sinusoidal current passed to the scalp. We tested whether alpha tACS would change somatosensory sensitivity or response bias in a signal detection task in order to test whether alpha oscillations have a causal role in behaviour. Active 10 Hz tACS or sham stimulation was applied using electrodes placed bilaterally at positions CP3 and CP4 of the 10–20 electrode placement system. Participants performed the Somatic Signal Detection Task (SSDT), in which they must detect brief somatosensory targets delivered at their detection threshold. These targets are sometimes accompanied by a light flash, which could also occur alone. Active tACS did not modulate sensitivity to targets but did modulate response criterion. Specifically, we found that active stimulation generally increased touch reporting rates, but particularly increased responding on light trials. Stimulation did not interact with the presence of touch, and thus increased both hits and false alarms. TACS stimulation increased reports of touch in a manner consistent with our observational reports, changing response bias, and consistent with a role for alpha activity in somatosensory detection.

## Highlights

• Active tACS stimulation alters response bias, not perceptual sensitivity.
• Both true and false reports of touch increase during active versus sham stimulation.
• Reports of touch increase more during stimulation when a light-flash occurs.

## 1 Introduction

There is a wide range of evidence across multiple sensory modalities that spontaneous, ongoing neural oscillations in the alpha band – 8–13 Hz – have a direct role in perception and determining which stimuli are detected and which missed (e.g., Busch et al., 2009, Chaumon and Busch, 2014, Craddock et al., 2017, Ergenoglu et al., 2004, Neuling et al., 2012). Much of this evidence is necessarily correlative, based on observations recorded using magneto- or electroencephalography (M/EEG). More direct evidence of causation requires direct manipulation of the ongoing oscillatory rhythms naturally and spontaneously exhibited by the brain.

Transcranial electrical stimulation (tES) offers one such method of directly influencing ongoing brain activity (Paulus, 2011). Three commonly used tES methods are transcranial direct current stimulation (tDCS), transcranial alternating current stimulation (tACS, Antal and Paulus, 2013), and transcranial random noise stimulation (tRNS). Of these, tACS is particularly promising as a method by which to interact with endogenous rhythms, since it allows application of a sinusoidal current at a desired frequency. Indeed, there are several reports that tACS stimulation at or around 10 Hz modulates alpha power, increasing it even after stimulation has ended (Helfrich et al., 2014, Vossen et al., 2015, Zaehle et al., 2010). Furthermore, modulation of alpha oscillations using tACS also influences detection of visual targets phasically (Helfrich et al., 2014), consistent with the pattern found previously in the absence of tACS stimulation (e.g., Mathewson et al., 2009, Mathewson et al., 2011, VanRullen et al., 2011).

Effects of tACS on other sensory modalities, including audition (Neuling et al., 2012) and pain (Arendsen et al., 2018), have been reported. Most relevant here, however, is how tACS stimulation may influence somatosensation. As in vision, tactile detection can vary with the power of alpha oscillations recorded over somatosensory regions. We found that detection of peri-threshold tactile stimuli was predicted from alpha power in a period shortly before stimulus onset (Craddock et al., 2017). In that study, participants performed the Somatic Signal Detection Task (SSDT; Lloyd et al., 2008), in which they were asked to detect brief somatosensory stimuli delivered to their left index finger at detection threshold. Brain activity was simultaneously recorded using EEG. We found that power in the alpha frequency band influenced both true and false reports of somatosensory perception. As pre-stimulus alpha power increased, the probability of reporting touch decreased, both in the presence and absence of target stimuli. Given that alpha plays a similar role in both visual and tactile detection, and that alpha tACS modulates visual detection, it follows that manipulation of alpha using tACS may also modulate somatosensory detection.

A study by Gundlach et al. (2016) found evidence consistent with this suggestion. They had participants perform a somatosensory detection task before, during, and after active alpha or sham tACS stimulation delivered over bilateral somatosensory cortices. Tactile stimuli were delivered to the participants' right index finger. The intensity of the stimuli was continuously varied, but maintained at detection threshold using a staircase procedure. Detection thresholds for the stimuli in the periods before, during, and after the stimulation period did not differ on average. However, during active stimulation, detection thresholds varied in a phasic manner. Detection thresholds at opposite phases of the driving oscillations differed from baseline (pre-stimulation) performance in opposing fashion: some phases were associated with decreased thresholds while others were associated with increased thresholds.

However, a limitation of Gundlach et al.’s (2016) study was that stimuli were always present. Thus, it is impossible to determine whether the changes in detection performance they observed were related to genuine variation in tactile sensitivity. TACS stimulation in the alpha frequency range may also induce faint tactile sensations contralateral to the stimulated region (Feurra et al., 2011), which might increase false reports of touch during stimulation. A typical way of assessing performance on detection tasks is to calculate signal detection measures (Macmillan and Creelman, 2005), which account for both hit rates – correct detection of target stimuli – and false alarm rates — false reports of target stimuli when the stimulus is absent. Sensitivity (*d*′) describes the ability to discriminate signal from noise. Response criterion (*c*) describes the degree of bias towards responding that a signal is present or absent.

In signal detection terms, the pattern of results reported in Craddock et al. (2017) is consistent with changes in response criterion rather than sensitivity, since alpha power shifted hit and false alarm rates in the same direction. In addition, Gundlach et al. (2017) reported that the somatosensory alpha rhythm decreased in power after tACS stimulation. Thus, in accordance with our results, decreases in power should increase reporting rates for touch, increasing both false alarms and hit rates, and thus not increase somatosensory sensitivity per se (Craddock et al., 2017). TACS stimulation might then change response criterion, biasing participants towards or against reporting stimuli, rather than changing sensitivity or detection threshold. Therefore, in order to test whether alpha tACS stimulation would induce changes in response bias, we had participants perform the SSDT while undergoing tACS.

## 2 Material and methods

### 2.1 Participants

Twenty-one right-handed participants (19 female, two male; ages: *μ* = 19.7 years, *σ* = .097) were recruited from the undergraduate population of the University of Leeds. Five additional participants were excluded following initial screenings for contraindications to receiving tACS stimulation (e.g. unremovable facial piercings, history of migraines). Participants received course credit or cash vouchers for participation. The study was approved by the ethical committee of the School of Psychology at the University of Leeds (ethics reference: 16-0019). All participants reported normal or corrected-to-normal vision and no tactile sensory deficits, and gave fully informed written consent.

### 2.2 Apparatus

The stimulus array comprised a soft foam block in which a piezoelectric tactile stimulator (PTS) was embedded (Dancer Design, St. Helens, UK), with a red light-emitting diode (LED) attached next to the PTS. Participants placed their left index finger on top of the PTS. Tactile stimuli were produced by an auditory signal delivered from the experimental PC to the tactile amplifier (TactAmp 4.2, Dancer Design). Note that vibrations from the PTS were entirely inaudible when it was embedded in the foam block. A monitor located behind the stimulus array delivered instructions and visual cues. Participants sat approximately 70 cm in front of the monitor, with the stimulus array to the left of their midline. Participants responded with a button box held in their right hand. Timing and presentation of the stimuli was controlled using EPrime 2.0.

#### 2.2.1 Transcranial alternating current stimulation (tACS)

Transcranial alternating current stimulation was applied using a neuroConn DC-Stimulator-Plus (Eldith, Neuroconn, Ilmenau, Germany). Two rubber electrodes (5 cm by 5 cm) in foam sponges – pre-soaked in saline solution – were placed over positions CP3 and CP4 of the international 10–20 electrode placement system. The sponges and electrodes were held in place using a rubber strap. Although impedances were not monitored during the experiment, the initial impedance could not exceed 5 kΩ.

### 2.3 Procedure

All participants took part in two experimental sessions separated by at least two days. Before beginning the experiment, the tACS montage was set up as above. The experiment itself was split into two parts. In the first part, each participant's sensory threshold (i.e., 50% detection rate) was established using a two-alternative forced choice adaptive staircase procedure. Participants were given a series of trials consisting of two consecutive 1420 ms time periods. Each time period began with a green arrow presented for 400 ms on the left side of the monitor and pointing down towards the participant's finger. The numbers “1” and “2” were written on arrows marking the start of the first and second periods respectively. After the offset of each arrow, the screen remained blank for 1020 ms. On each trial, a 20 ms tactile pulse was delivered 500 ms after the offset of either the first or second arrow. After both time periods had elapsed, participants were prompted on screen to press button 1 or 2 on the button box to report whether the stimulus had been presented in the first or second time period. A further 1000 ms elapsed before the start of a new trial. Trials were repeated until a stable 50% detection threshold was reached or up to a maximum of 150 repetitions (no participant exceeded this maximum). Participants did not receive feedback.

In the main experiment, participants were asked to detect brief 20 ms tactile pulses delivered at sensory threshold. In the sham condition, random noise stimulation was applied for 30 s at 1.5 milliamps (mA). In the active condition, a 10 Hz alternating current was delivered at 1.5 mA for 25 min(the approximate length of the experiment). The order of stimulation conditions was counterbalanced across subjects. In both conditions, stimulation ramped up from zero to 1.5 mA over 30 s at the beginning, and sloped back down to zero over 10 s at the end. At the start of each trial, a green arrow pointing down towards the participant's left index finger appeared for 500 ms. This was replaced with a blank screen for 1 to 1.5 s, which was followed by a 20 ms stimulus period.

There were four possibilities in the stimulus period: a touch delivered alone, a light flash alone, a touch and light flash delivered simultaneously, or neither a touch nor a light flash. Each of these occurred on a quarter of trials. There were 204 trials in total Thus, each of the four trial types – touch alone, light alone, both light and touch, and no stimulus – occurred 51 times. After the 20 ms stimulus period, there was a further 750 ms of blank screen. Finally, a response screen appeared asking the participant if they had felt a touch. Participants were asked to respond using the button box held in their right hand with one of four buttons to indicate “Definitely yes”, “Maybe yes”, “Maybe no”, or “Definitely no”. The response screen disappeared when the response was made. No feedback was provided. Finally, the screen remained blank for 1 to 1.5 s before the next trial.

### 2.4 Data analysis

We first performed three analyses using a standard ANOVA framework. These analyses were performed primarily for comparison with previous studies using the SSDT, which used standard ANOVA analyses of touch reporting rates and of the signal detection measures sensitivity (*d*′) and response criterion (*c*). For all analyses, we combined “Definitely yes” and “Maybe yes” into “yes” reports and “Definitely no” and “Maybe no” into “no” reports.

For the analysis of Type-I signal detection measures, we calculated *d*′ and *c* separately for trials with and without a light, and during active and sham stimulation. “Yes” reports on touch trials were hits; “yes” reports on no touch trials were false alarms. “No” reports on touch trials were misses; “no” reports on no touch trials were correct rejections. Thus, we had four *d*′ and four *c* measures for each participant. The log-linear correction was used to account for cells with either 100% or 0% reports of touch. For the analysis of reporting rates, we ran a repeated-measures ANOVA with the factors Touch (Touch/No touch), Light (Light/No light), and Stimulation (Active/Sham) with the percentage of reports of touch as the dependent variable. Where necessary, post-hoc *t*-tests with Bonferroni-Holm correction for multiple comparisons were conducted to decompose significant interactions.

In addition to our standard ANOVA analyses, we also fitted a Bayesian generalized linear mixed effects model using the *brms* package (see below). Mixed-effects models are extensions of standard general linear regression models that allow simultaneous modelling of both fixed and random effects. When multiple observations are recorded from individual groups, they yield correlated observations that would violate the assumption of independence necessary to model them using a standard fixed-effects model. Simultaneously modelling random effects allows the correlation structure to be appropriately incorporated into the model.

In addition, using a generalized linear model allows us to appropriately model data that does not follow a normal distribution. For this analysis, we combined “Definitely yes” and “Maybe yes” into “yes” reports and “Definitely no” and “Maybe no” responses into “no” reports. We then coded “yes” responses as 1 and “no” responses as 0, and thus, our outcome for each trial was binary. As mean reporting rates approach 100% or 0%, the variance decreases. ANOVA conducted on percentages does not account for such changes in variance and can lead to misleading conclusions (Jaeger, 2008). We therefore chose to use a logistic link function, and thus a logistic regression model or our data.

#### 2.4.1 Generalized linear mixed effects models

For further clarification, first, we describe the relationship between a standard linear model and a generalized linear model. A standard general linear model is given by the equation:

y=Xb+e

*y* is the response variable. *X* is a matrix of linear predictors or independent variables. *b* is vector of the unknown effects of those predictors, which are the quantities to be estimated by the model fitting process. The effects – typically called model coefficients – are interpretable as the change in the response variable resulting from a one unit change in the predictor variable. Finally, *e* is a normally distributed error term (*N*∼ (0, *σ*^2^)). Note that we could expand them equation out to show individual terms, e.g.

$$y=\beta_{0}+\beta_{j}+\epsilon$$

where *β*_0_ is the intercept – or the grand average across all conditions – and *β*_*j*_ are individual coefficients for each of *j* terms in the model.

In the above formulation, we are assuming only fixed effects. However, to appropriately capture the hierarchical structure, we have to also model how effects vary across participants. If *i*∈ {1, …, *n*} where *n* = the number of participants, then we can expand the model as follows:

$$y_{i}=X_{i}b+Z_{i}v_{i}+e_{i}$$

*y*^i is now the response vector for each individual participant. *X* is the design matrix for the fixed effects expanded across all participants, and *b* a vector of fixed effects coefficients. Note that these are not participant-specific. *Z* is the random effects design matrix, *v* is the vector of random effects coefficients. These are participant-specific estimates of the random effects. In this formulation, all effects are modelled as varying across participants.

However, as noted above, our response variable is binomial, and the model errors cannot be assumed to be normal. We thus need to adapt the model further. Generalized linear models replace the assumption of a normally distributed error term with a *link* function that transforms the model residuals, allowing, for example, modelling of data where the variance depends on the mean:

$$E(y)=g^{-1}Xb$$

Here, *g*^−1^ represents the link function, while *E*(*y*) represents the expected value (i.e. mean) of *y*. When the data to be modelled is binomial, we are thus modelling the probability of *y* taking on the value 1 as a function of the predictors.

A natural way to express the probability of an outcome is in terms of odds, which are derived from probability *p* of a given outcome occurring as $odds=\left(\frac{p}{1-p}\right)$. Odds increase as the probability of a given event increases. However, odds cannot be linearly combined, and are typically transformed to the log-odds or *logit* scale by taking their natural logarithm — $logit(p)=\ln\left(\frac{\hat{p}}{1-\hat{p}}\right)$ where $\hat{p}$ is the expected proportional response. Thus, what is modelled is the proportional response as a function of design matrix *X* and the vector of coefficients *b*. Note that this means expected proportions are expressed in logits, and the coefficients are expressed as the change in logits.

If we now adapt the mixed-effects model equation given above, we have a generalized linear mixed models with a logistic link function:

$$logit(p)=X_{i}b+Z_{i}v_{i}$$

This model properly accounts for both the binomial nature of the response variable and the hierarchical structure of the data. After model fitting, the fixed-effects coefficients, *b*, are thus changes in logits as a function of the predictors given in design matrix *X*, and marginalized over the participant-specific coefficients, *v*. These coefficients have the same interpretation as in standard GLMs, in that they represent the logit change in the response variable as a result of a one unit change in the predictor. However, they are difficult to intuitively interpret, since they are on a very different scale from the original response variable. A common approach is to exponentiate the logit coefficient, which returns an *odds ratio*. Odds-ratios have a much more intuitive interpretation. For example, an odds ratio of 2 for a given predictor indicates that increasing that predictor by 1 unit makes a response twice as likely.

#### 2.4.2 Specification and interpretation of the Bayesian GLMM

The model contained three fixed effects factors – Stimulation (Active or Sham), Touch (Touch or No touch), and Light (Light or No light) – and all interactions between them. Participant was specified as a random effect, with random slopes for each fixed effect and all interactions, random intercepts, and a full, unstructured correlation matrix. The model was fit using an adaptive Hamiltonian Monte-Carlo Markov-Chain (MCMC) algorithm implemented in Stan (Carpenter et al., 2017). In brief, this process estimates regression coefficients through exploration of the parameter space to produce a posterior distribution. The posterior distribution thus represents plausible parameter values after taking into account both our priors and the data we observed. Here, we use the mean of the posterior distribution for each parameter as the estimated *β* coefficient for that parameter. We additionally summarise each distribution using 95% credible intervals, which capture 95% of the posterior probability mass. To further aid interpretation, we also report the posterior probability as the proportion of the posterior distribution that is below zero. The higher this proportion, the more likely it is that a particular parameter is above zero under this model, and vice versa. Thus, a proportion of .5 would indicate that parameter is equally likely to above or below zero.

We used non-informative priors in our analysis. Specifically, there were improper uniform priors from negative to positive infinity on the mean for population-average (i.e. fixed) effects, including the intercepts; an LKJ-prior (*ν* = 1) on the correlations between the random slopes and the intercept; and a half (i.e. constrained to be positive) Student-*t* prior with shape parameter 3 and scale parameter 10 on the standard deviations of the random slopes. These priors provide little information regarding the parameter values, primarily serving to regularize the estimates of the parameters of the random effects structure. This ensures that all parameters are identifiable, and biases them against reaching improbably large values. We ran four Markov chains simultaneously, each for 5000 iterations. The first 2500 of those iterations were discarded as warm-up samples to adaptively tune the MCMC sampler. Convergence of the chains was assessed by visual inspection of their traces, which indicated that they mixed well and converged on the same parameter spaces. The $\hat{R}$ statistic (Gelman and Rubin, 1992) was ~1.00 for all parameters.

All analyses were conducted using R (Version 3.5.1; R Core Team, 2018) and the R-packages *afex* (Version 0.22.1; Singmann et al., 2018), *brms* (Version 2.6.0; Bürkner, 2017), *emmeans* (Version 1.3.0; Lenth, 2018), *metaSDT* (Version 0.5.0; Craddock, 2018), *papaja* (Version 0.1.0.9842; Aust and Barth, 2018), *tidybayes* (Version 1.0.3; Kay, 2018), and *tidyverse* (Version 1.2.1; Wickham, 2017).

## 3 Results

### 3.1 Standard ANOVA analyses

**Fig. 1:**
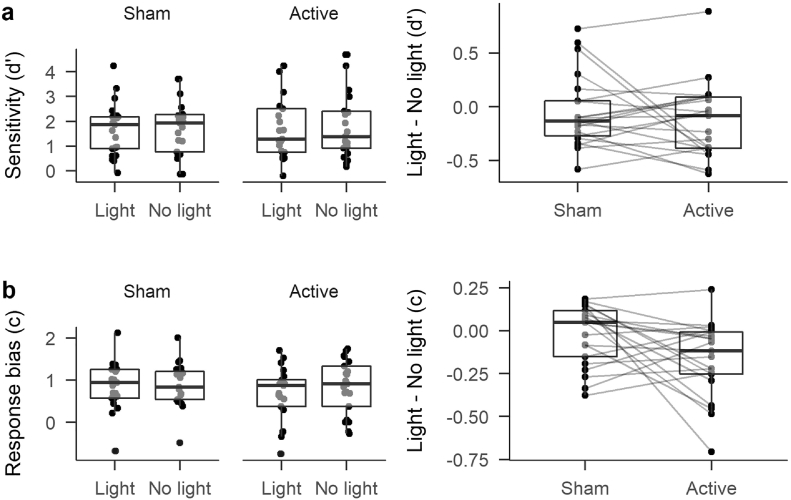
Boxplots of the signal detection measures d′ (row a) and c (row b). Boxes indicate the inter-quartile range. Lines within the boxes indicate the median. Whiskers extend 1.5 times above and below the inter-quartile range. Individual dots show individual participant scores. The right column shows the difference between d′ and c in the Light and No Light conditions in order to show the interaction between light and stimulation. Lines connecting individual dots join data points belonging to the same participant.

We first examined performance in a classical SDT framework. We found no significant difference in sensitivity (*d*${}^{\prime }$) between trials with a light (1.70) and trials without a light (1.79, [*F*(1,20) = 2.07, *MSE* = 0.08, *p* = .165, ${\hat{\eta }}_{G}^{2}=.001$]), and no significant effect of Stimulation on *d*′ [Sham = 1.69; Active = 1.80; *F*(1,20) = 0.16, *MSE* = 1.59, *p* = .693, ${\hat{\eta }}_{G}^{2}=.002$]. There was also no significant interaction between Stimulation and Light on *d*′ [*F*(1,20) = 1.04, *MSE* = 0.04, *p* = .319, ${\hat{\eta }}_{G}^{2}=.000$], see Fig. 1a.

For response criterion (*c*), there was no significant main effect of Stimulation (Sham = 0.89; Active = 0.76; [*F*(1,20) = 1.02, *MSE* = 0.35, *p* = .325, ${\hat{\eta }}_{G}^{2}=.012$]). However, there was a significant main effect of Light [*F*(1,20) = 10.03, *MSE* = 0.02, *p* = .005, ${\hat{\eta }}_{G}^{2}=.006$], with a more liberal bias (i.e. an increase in “yes” reports) on light trials (*c* = 0.77) than on no light trials (*c* = 0.87). Importantly, there was a significant interaction between Stimulation and Light [*F*(1,20) = 5.16, *MSE* = 0.02, *p* = .034, ${\hat{\eta }}_{G}^{2}=.004$], see Fig. 1b. This interaction was driven by a significant difference between light and no-light trials in the Active stimulation condition (*p* = .001, Bonferroni-Holm corrected for 6 comparisons). Specifically, there was lower *c* on trials with a light (*c* = 0.67) than on trials with no light (*c* = 0.84). In Fig. 1b, the pattern of lines in the interaction plot suggest a degree of heterogeneity in the interaction between Stimulation and Light for response criterion, but with the most consistent change being a shift towards a more liberal response criterion for light trials relative to no light trials (i.e. more negative values). No other comparisons were significant (all *p*s = 1) .

**Fig. 2:**
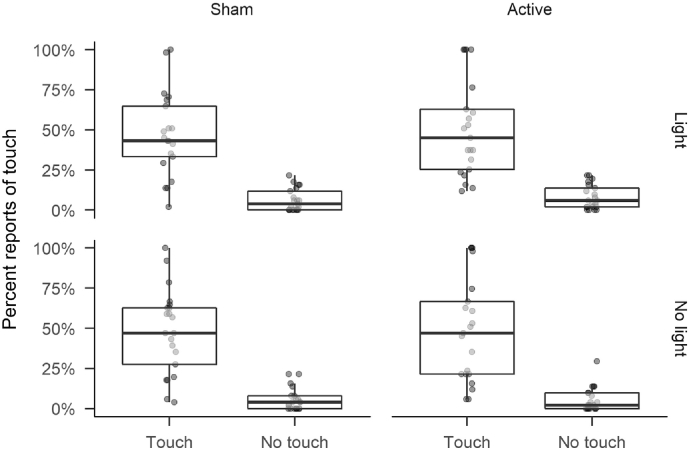
Boxplots of mean response rates in each combination of stimulation, touch and light conditions. Boxes indicate the inter-quartile range. Whiskers extend 1.5 times above and below the limits of the inter-quartile range. Lines within the boxes show the median. Individual dots indicate mean response rates for individual participants.

In our analysis of reporting rates, there was a significant effect of Touch [*F*(1,20) = 57.56, *MSE* = 0.13, *p* < .001, ${\hat{\eta }}_{G}^{2}=.516$], with reports of touch much more likely on trials with touches (48.51%) than without (6.26%). No other effects were significant (all *p*s > .06; see Table 1, Fig. 2).

**Table 1:** Results of the repeated measures ANOVA on touch reporting rates.

Effect	*F*	*df*_1_	*df*_2_	*MSE*	*p*	${\hat{\eta }}_{G}^{2}$
Touch	57.56	1	20	0.13	<.001	.516
Light	1.78	1	20	0.00	.197	.001
Stimulation	0.22	1	20	0.04	.644	.001
Touch × Light	0.52	1	20	0.00	.479	.000
Touch × Stimulation	0.04	1	20	0.05	.844	.000
Light × Stimulation	3.99	1	20	0.00	.060	.001
Touch × Light × Stimulation	0.19	1	20	0.00	.666	.000

### 3.2 Bayesian multilevel model

**Fig. 3:**
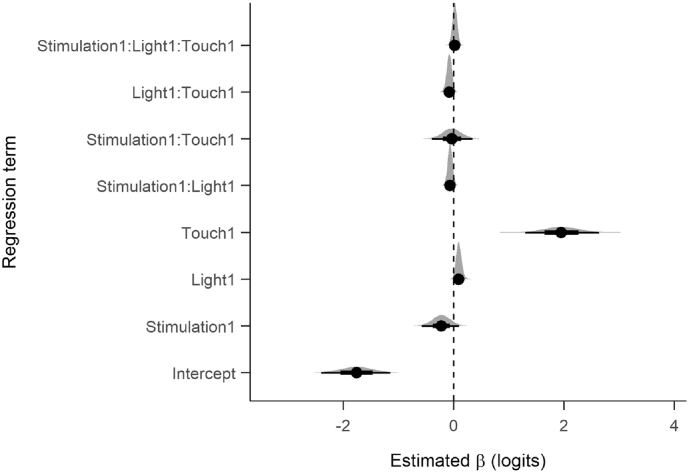
Posterior densities and credible intervals for the fixed effect coefficients. Dots indicate the mean of the posterior distribution. Bars indicate 66% (thick) and 95% (thin) credible intervals.

**Fig. 4:**
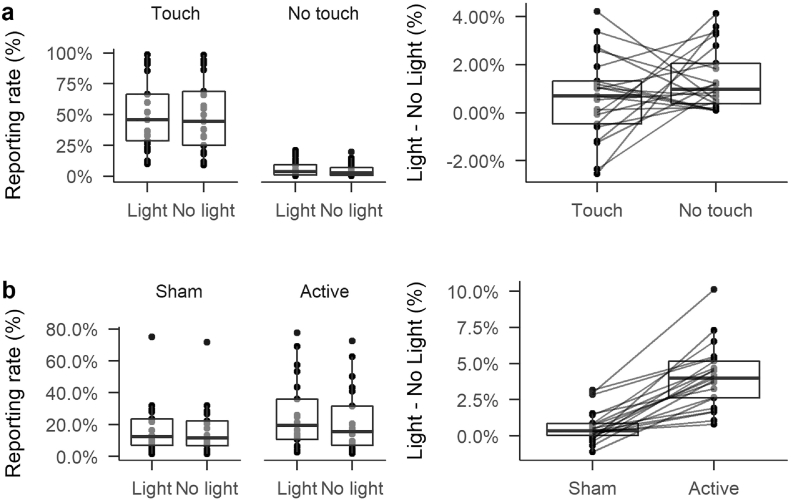
Boxplots showing model predicted yes-response rates (left) and the percentage point difference in yes-response between Light and No light trials (right). Boxplots span the inter-quartile range of the data, with the median shown by a single line. Whiskers extend 1.5 times the IQR above and below the hinges of the boxes. Each dot represents predicted values for individual participants. Lines join predictions from individual participants.

The Bayesian GLMM proved notably different from the repeated measures ANOVA on reporting rates (see Table 2, Fig. 3). The Bayesian *R*^2^ for the model was .429 (Gelman et al., 2017). The strong effect of Touch on reporting rates was consistent with the ANOVA, but the model also suggests that there was a small increase in reporting rates on Light trials, with the vast majority of posterior samples for this coefficient being above zero (*p*(*β* < 0) = 0.02). Furthermore, the interaction between Light and Touch was also strongly likely to be negative (*p*(*β* < 0) = 0.96). On touch trials, the difference between light and no light trials was inconsistent, sometimes positive, sometimes negative. On no touch trials, reporting rates were consistently higher on light trials than on no light trials (see Fig. 4a).

**Table 2:** Table of fixed effects from the Bayesian GLMM.

Term	Beta	SE	Lower CI	Upper CI
Intercept	0.17	1.37	0.09	0.32
Stimulation1	0.80	1.18	0.56	1.10
Light1	1.10	1.05	1.01	1.22
Touch1	7.08	1.40	3.66	13.95
Stimulation1:Light1	0.94	1.05	0.86	1.02
Stimulation1:Touch1	0.97	1.20	0.68	1.40
Light1:Touch1	0.93	1.05	0.84	1.01
Stimulation1:Light1:Touch1	1.02	1.04	0.94	1.12

*Note.* CIs are 95% credible intervals. All units are odds-ratios.

More importantly, the model also suggested that some Stimulation effects were also non-zero. The coefficient for the effect of Stimulation was negative (*β* = 0.80), and most of the posterior density fell below 0 (*p*(*β* < 0) = 0.91), indicating that the coefficient has a high probability of being below zero. Thus, reporting rates were likely higher overall in the Active condition than in the Sham condition. Importantly, the interaction between Stimulation and Light, though small, was also likely to be negative (*β* = 0.94, CIs = [0.86, 1.02], *p*(*β* < 0) = 0.92). As can be seen in Fig. 4b, on Sham stimulation trials, there was little difference between trials with a light and without a light. But during Active stimulation, all participants showed increased reporting of touches during trials with a light compared to trials without a light.

Critically, there was little evidence of an interaction between Stimulation and Touch. The posterior density spanned zero, with only a low probability of the parameter being negative (*p*(*β* < 0) = 0.57). The three-way interaction between Stimulation, Touch, and Light was similarly equivocal, albeit with a posterior probability more in favour of the parameter being positive than negative (*p*(*β* < 0) = 0.29). Thus, to the extent that Stimulation had effects on reporting of touch, these effects were driven by changes in responses to the light.

## 4 Discussion

We examined the effects of 10 Hz transcranial alternating current stimulation (tACS) over centro-parietal regions on performance of the Somatic Signal Detection Task. We previously reported that oscillatory activity in this frequency range influenced reporting of touch independently of whether touch is actually present (Craddock et al., 2017). Our analysis of signal detection measures suggested that tACS stimulation did not influence detection sensitivity, but did introduce a more liberal bias towards responding that touch was present, especially when the light was present. Our Bayesian model also suggests that reports of touch were increased during active stimulation, with an additional increase in the presence of light flashes. This was independent of whether a target touch stimulus was present or not. In combination, these results suggest that tACS stimulation at 10 Hz modulated response bias independently of sensitivity.

We reported in Craddock et al. (2017) that reports of touch decline as pre-stimulus alpha power increases and increase as it decreases, independent of whether touch is present. This would imply that tACS would need to decrease alpha power in order to increase reports of touch. For visual alpha, oscillatory power during stimulation shows an online increase during stimulation (e.g. Helfrich et al., 2014). This increase persists after stimulation (e.g. Kasten et al., 2016, Vossen et al., 2015, Zaehle et al., 2010), and thus the offline effects mirror the online effects of stimulation. Gundlach et al. (2017) reported a decrease in the power of somatosensory alpha rhythms after 10 Hz tACS stimulation, but online effects of tACS stimulation on somatosensory alpha are as yet unclear. If online effects of 10 Hz tACS on somatosensory alpha mirror offline effects, as in vision, they would lead to an increase in reporting of touch and a more liberal response bias, as found here.

There are grounds to suggest that the stimulation montage itself may influence the direction of changes in alpha power. With tACS, the direction of current flow alternates between electrodes, so a peak at the left electrode would be mirrored by a trough at the right electrode and vice versa. A typical anterior-posterior montage used for stimulating parieto-occipital alpha would produce phasic stimulation of the alpha generators, whereas the non-central montage we used and that used by Gundlach et al., stimulate bilateral somatosensory cortices in antiphase. A computational study by Kutchko and Fröhlich (2013) suggested that phasic stimulation across multiple network sites should increase oscillatory power and enhance synchronization between those sites, whereas antiphasic stimulation would disrupt synchronization and not increase in oscillatory power. Nevertheless, non-central stimulation has been used over occipital regions in the study of visual alpha, which resulted in the typical increase in alpha power (Vossen et al., 2015, Zaehle et al., 2010). Thus, another possibility is that the differences in the direction of alpha power changes across somatosensory and visual tACS studies may be driven by different dynamics in the targeted brain regions, rather than differences in stimulation montage per se.

An explanation for the influence of alpha power on touch is that it may reflect variation in cortical excitability (e.g., Iemi et al., 2016, Kelly et al., 2006, Lange et al., 2013, Romei et al., 2008). Alpha power increases as cortical inhibition increases, and decreases with increased cortical excitability (Peterson and Voytek, 2017). The balance of excitation and inhibition across cortical areas may reflect suppression of sensory responses during selective attention (Foxe and Snyder, 2011). For example, during visual spatial attention tasks, oscillatory power in the alpha band is lower over the hemisphere contralateral to the attended region of space and higher over the hemisphere ipsilateral to the ignored region of space (e.g. Thut et al., 2006). Increasing inhibition suppresses low-level cortical responses and restricts outflow of information to higher-level cortical areas (Jensen and Mazaheri, 2010). Concomitantly, an increase in excitability lifts that gate and allows more information out, therefore shifting to a more liberal response bias.

Nevertheless, in the context of an increase in cortical excitability in somatosensory cortex, the interaction with the light is unexpected. We might instead have expected overall response rates to increase irrespective of the influence of the light. However, the effect of light was multiplicative with active stimulation. Active stimulation increased reports of touch even without the light; the increase was simply larger when the two were combined. During sham stimulation, there was little consistent difference in reporting rates between light and no-light trials. Thus, the combination of both active stimulation and light flashes induced a more liberal response bias. An increase in output from somatosensory regions would give increased opportunities for the light to boost responses to perceived somatosensory stimulation. More broadly, since regions beyond our putative target processes and oscillatory rhythms in somatosensory cortex also play a role in the decision making process, we must also consider the possibility that our stimulation influenced these additional, non-target regions. A primary candidate would be motor cortex. However, current evidence suggests tACS at 10 Hz has little impact on motor cortex, with stimulation at 20 Hz, in the beta frequency range, having a much stronger influence (Feurra et al., (2011), Feurra et al., (2013)). Other studies of more abstract decision making typically involve stimulation with different montages. More plausibly, regions involved in supramodal processes, such as parietal cortex (Levine and Schwarzbach, 2017), may have been affected by our stimulation. This may have lead to a reweighting of sensory evidence towards greater use of the visual signal.

Our results do come with some caveats. First, our comparison of active versus sham stimulation would not allow us to make concrete statements about the specificity of stimulation at a particular frequency, since we stimulated only at a single frequency. Second, since we did not record EEG before and after stimulation, we cannot be sure that we directly influenced visual alpha or somatosensory alpha rhythms. Finally, since we used only a single pair of stimulation locations, we cannot necessarily distinguish between non-specific effects of tACS and direct effects of stimulation on the specific rhythms of interest. Overall, however, our results are consistent with tACS stimulation at 10 Hz over somatosensory regions altering response bias in the SSDT, and thus provide support for a direct role of alpha oscillatory rhythms in tactile perception.
